# Screening for protein-protein interactions using Förster resonance energy transfer (FRET) and fluorescence lifetime imaging microscopy (FLIM)

**DOI:** 10.1038/srep28186

**Published:** 2016-06-24

**Authors:** Anca Margineanu, Jia Jia Chan, Douglas J. Kelly, Sean C. Warren, Delphine Flatters, Sunil Kumar, Matilda Katan, Christopher W. Dunsby, Paul M. W. French

**Affiliations:** 1 Imperial College London, Dept. Physics, Photonics Lab., Blackett building, Prince Consort Road, London, SW7 2AZ, UK; 2 University College London, Institute of Structural and Molecular Biology, Darwin building, Gower St., London, WC1E 6BT, UK; 3 Imperial College London, Institute of Chemical Biology, London, SW7 2AZ, London, UK; 4 Université Paris Diderot, Sorbonne Paris Cité, Molécules Thérapeutiques in silico, Inserm UMR-S 973, 35 rue Helene Brion, 75013 Paris, France

## Abstract

We present a high content multiwell plate cell-based assay approach to quantify protein interactions directly in cells using Förster resonance energy transfer (FRET) read out by automated fluorescence lifetime imaging (FLIM). Automated FLIM is implemented using wide-field time-gated detection, typically requiring only 10 s per field of view (FOV). Averaging over biological, thermal and shot noise with 100’s to 1000’s of FOV enables unbiased quantitative analysis with high statistical power. Plotting average donor lifetime vs. acceptor/donor intensity ratio clearly identifies protein interactions and fitting to double exponential donor decay models provides estimates of interacting population fractions that, with calibrated donor and acceptor fluorescence intensities, can yield dissociation constants. We demonstrate the application to identify binding partners of MST1 kinase and estimate interaction strength among the members of the RASSF protein family, which have important roles in apoptosis via the Hippo signalling pathway. *K*_*D*_ values broadly agree with published biochemical measurements.

With increasing knowledge of intracellular signalling networks, it becomes more evident that molecules can be involved in processes occurring in multiple pathways. Understanding the complex interconnections between different pathways requires comprehensive identification of specific binding partners, and therefore it is important to develop higher throughput techniques to search for new interactions. Currently, biochemical methods are most often used to this end and provide high sensitivity. However, they require long separation procedures, during which the active molecules are isolated from their native environment and may present different reaction kinetics than in live cells where molecular crowding and high compartmentalisation could have an impact. Fluorescence microscopy – particularly exploiting genetically expressed fluorescent proteins – can be applied directly to map and quantify protein interactions in live or fixed cells and preserve information concerning the inhomogeneous cellular distribution of molecules, with typical spatial resolution below 0.5 μm. With the advent of superresolution microscopy, the prospect of sub-50 nm resolution could permit the study of the organisation and dynamics of molecules within organelles and large interacting complexes^1^,^2^. However, manual fluorescence microscopy experiments are subject to operator bias and it is impractical to undertake measurements on a sufficient number of cells to identify systematic errors and to average over “biological noise”. Large scale screening using automated fluorescence microscopes can provide higher throughput studies of signalling processes with improved statistical significance. To date, high content analysis platforms for cell imaging have been mostly based on fluorescence intensity readouts and have predominantly been applied to study the effects of inhibitors on signalling pathways^3^. Other fluorescence parameters may also be utilised to assay molecular environment (fluorescence lifetime) or fluorophore orientation (polarisation/anisotropy).

A widely used fluorescence technique to study bi-molecular interactions within cells is FRET (Förster resonant energy transfer), which utilises the non-radiative (dipole-dipole) energy transfer from a fluorescent donor to an acceptor that can take place only when the two fluorophores are situated at distances <~10 nm. In the case of two proteins labelled with donor and acceptor tags, this implies that FRET occurs only if and when the two proteins interact with each other. FRET has therefore been widely exploited to study protein interactions using fluorescence microscopes. However, its application for high content analysis in automated multiwell plate readers is much more limited. FRET can be read out using a wide range of techniques^4^ although most of these are not practical for rapid automated assays of multiwell plates where hundreds to thousands of fields of view are imaged in a single experiment.

One approach to detect FRET is to measure the fluorescence intensity ratio of the acceptor and the donor fluorophores observing the increase of the fluorescence intensity in the acceptor channel with the simultaneous decrease of the intensity in the donor channel. This spectral ratiometric imaging acquisition is fast but requires additional control samples to correct for spectral cross-talk between the fluorophores and to calibrate the spectral response of the specific optical set-up (instrument and sample corrections), making comparison between different samples difficult. Quantitation can be degraded by unknown variations in donor-acceptor stoichiometry and quantitative readouts of FRET efficiency and population fraction of FRETing donors are not possible without additional measurements of reference FRET constructs^5^,^6^.

It is also possible to utilise the depolarisation of the acceptor fluorescence as a FRET readout Polarisation-based measurements can achieve similar acquisition speeds as spectral ratiometric readouts and are highly sensitive to detect the occurrence of FRET, but it is again difficult to quantify FRET efficiencies and population fractions of interacting donors^7^,^8^. Polarisation has been applied as a first step to screen for possible interaction partners that were subsequently investigated using fluorescence lifetime^9^.

Fluorescence lifetime imaging (FLIM)^10^ provides a more robust approach to reading out FRET since only measurements of the donor fluorophores are required – negating the need for spectral calibration and providing readouts that can be directly compared between instruments and which can be translated from cell-based assays to animal models^11^. Compared to spectral or polarisation ratiometric techniques, FLIM requires more detected photons to achieve a given accuracy, so this is a slower modality for mapping and quantifying FRET in high content analysis. However, FLIM can also provide more quantitative readouts in a single spectral channel since the fluorescence decay profiles can be fitted to complex models in order to obtain the FRET efficiency and the interacting population fraction. Time-resolved measurements can be extended to analyse homoFRET and polarisation anisotropy decays using appropriate models. Fitting lifetime data to complex models typically requires 10,000’s of photons – compared to ~200 photons required to fit to a monoexponential decay model^12^ – and it is not possible to detect such high photon numbers per pixel from biological samples such as cells labelled with fluorescent proteins before photobleaching and phototoxicity ensue. However, global analysis techniques that fit data simultaneously from many pixels can overcome this limitation (subject to assumptions about spatial invariance of lifetime components across the data set) and enable FLIM data with only 100’s photons/pixel (i.e. compatible with live cell imaging) to be fitted to complex decay models. Quantitative information can also be directly obtained without fitting using the phasor analysis approach^13^,^14^.

In this paper we report the application of a prototype high content assay platform providing unsupervised FLIM FRET of multiwell plate arrays that can identify protein binding partners in their cellular context and quantify the dissociation constant, *K*_*D*_. In order to achieve the fast FLIM acquisition required for reading 96-well plates, we utilise wide-field time-gated imaging to realise a FLIM microscope that is able to automatically acquire wide-field or optically sectioned fluorescence lifetime images with a typical mean acquisition time of 10 seconds per field of view for cells expressing fluorescent proteins, including the time required to move from the previous field of view and to automatically focus the microscope. For the first time to our knowledge, we report the application of automated FLIM FRET to screen for protein binding partners within cells – here shown to identify interactions between the Ras-association domain family (RASSF) and mammalian sterile 20-like kinases (MST) – and the estimation of the *K*_*D*_ for these interactions.

The RASSF family consists of ten members, RASSF1-10, which share a common Ras association domain. The role of this domain is not yet fully understood^15^, but the RASSF proteins are components of the MST/Hippo pathway, which is considered to restrict cell proliferation, thus they potentially play important roles in suppressing tumourigenesis^16^,^17^,^18^,^19^,^20^,^21^,^22^. This could oppose the Raf/Mek/Erk stimulation of cell growth/proliferation that also depends on Ras activation (Fig. 1). More recently, RASSF1 and MST1 have also been shown to influence the cardiac function in response to stress^23^, whilst RASSF5 and MST1 are involved in mediating TNFα- and TRAIL-induced apoptosis^24^.

The C-terminal domains differ between the classical RASSF1-6 and the N-terminal RASSF7-10. The classical RASSF members have a common α-helical SARAH domain that is absent in the N-terminal RASSF members, which instead are predicted to have coiled-coil motifs at various positions towards their C-terminal region^25^. The SARAH domain, whose name is essentially derived from the three proteins that share this common feature at their extreme C-terminal region: Salvador/RASSF/Hippo^16^ is also found in the MST kinases. The mammalian homologues, WW45, RASSF and MST respectively, are components of the well conserved Hippo signalling pathway, which was first described in *Drosophila melanogaster*.

Recent studies have shown that the SARAH domain is able to dimerise in solution^26^,^27^,^28^, so it was hypothesized that dimerisation could represent a key mechanism of interaction between the MST kinases and RASSF proteins. It is thought that, through this dimerisation, RASSFs are involved in the regulation of the catalytic activity of MST kinases (Fig. 1). Being themselves devoid of enzymatic activity, RASSF proteins may act as scaffolds binding the MST kinases. Previous studies have shown interaction between several RASSF members and different Ras proteins^15^,^29^,^30^,^31^, leading to the hypothesis that Ras association localizes RASSFs and the MST kinases to the cell membrane, thus bringing the MST kinase domains into close proximity for trans-activating phosphorylation, driving the MST/Hippo pathway and cellular apoptosis^17^,^18^,^32^.

The SARAH dimer is formed via a head-to-tail interaction of the two helices in an antiparallel arrangement. Although different coiled-coil motifs have been described to form oligomers^33^,^34^, there is no evidence thus far to suggest that the predicted coiled-coils in the N-terminal RASSF7-10 are capable of associating with the SARAH domain of MST to promote their interactions. This study aimed to confirm at the cellular level that SARAH-mediated dimerisation is the mode of interaction by identifying the RASSF proteins that associate with MST1 kinase or its isolated SARAH domain. In addition, point mutations were also introduced within the SARAH domains of the more well-studied members, RASSF1 and 5, to study their effect on the dimerisation with the MST1-SARAH domain, which were assayed using FRET.

## Results and Discussions

**Figure 2:**
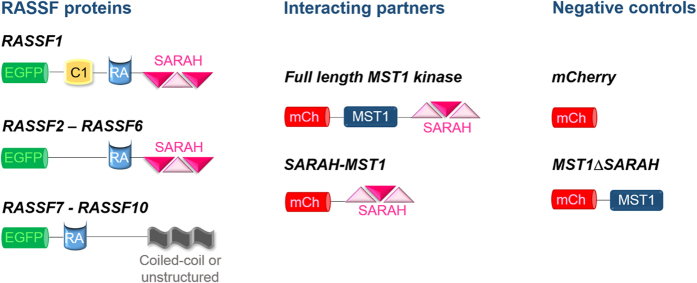
Schematic representation of the fluorescent constructs used for the FRET assays. The domain structure of the RASSF family members, of their possible interacting partners (MST1 kinase and its isolated SARAH_MST1_ domain) and of the negative controls are shown.

Figure 2 illustrates the fluorescent constructs that have been created to assay the RASSF-MST interactions using FRET. All ten RASSF proteins have been modified by attaching the fluorescent protein EGFP to their N-terminus to serve as a donor. Similarly, the MST1 kinase and its isolated SARAH domain (SARAH_MST1_) were labelled with mCherry at the N-terminus to provide the acceptor for the FRET assays. To evaluate the effect of possible non-specific interactions on the FRET readouts (e.g. arising from high local concentration of donor and acceptor), two negative controls were employed: the free fluorescent protein mCherry was expressed (without being linked to the MST1 or SARAH_MST1_ domain) and, as a more biologically relevant control, the kinase domain of the MST1 with a deletion of the SARAH domain (MST1ΔSARAH) was tagged with mCherry at its N-terminus.

**Figure 3:**
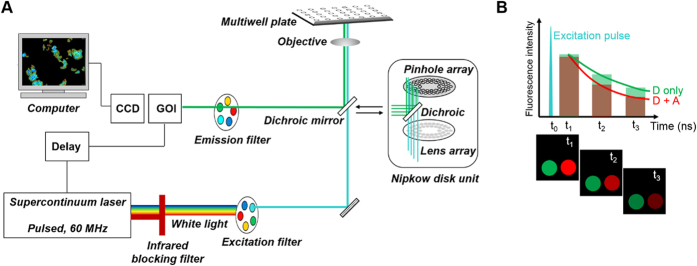
Schematic of automated plate reader based on time-gated fluorescence lifetime imaging (FLIM). (**A**) The pulsed excitation light is selected with an appropriate filter from the “white light” emitted by an ultrafast supercontinuum laser source and enters the microscope either in a wide-field configuration or via a Nipkow disk unit to provide optical sectioning. The fluorescence is detected via a gated optical intensifier (GOI) that acts as a fast (~100 ps rise time) electronic shutter synchronised with the laser pulses. The GOI opens at various delays after excitation (e.g. t_1_, t_2_, t_3_) and intensity images are acquired with a CCD camera at each time delay, integrating for a few seconds. (**B**) Lifetime determination. The time-gated images (t_1_, t_2_, t_3_) are used to reconstruct the fluorescence decay of the fluorophore, which is analysed by fitting exponential decay functions, discriminating between the lifetime of the donor only (D only) and the lifetime of the donor undergoing FRET in the presence of the acceptor (D + A).

Figure 3 shows a diagram of the automated FLIM multiwell plate microscope that can be configured for wide-field imaging or for optical sectioning using a Nipkow spinning disk unit, the latter providing more quantitative readouts at the cost of increased complexity. FLIM is realised using a gated optical intensifier that acts as a fast (~100 ps rise time) electronic shutter synchronised with the laser pulses, opening at various delays after excitation to provide time-gated fluorescence intensity images for each time delay, integrated over a few seconds. From these images, the fluorescence decay profiles can be reconstructed and analysed by fitting to an appropriate exponential decay model.

To obtain reliable statistics, we automatically acquired FLIM images from 10 fields of view (FOV) per well, using 5 time gates to sample the fluorescence decay profiles with exposure times around 1 s per gate for the donor (EGFP) images. Intensity images of the acceptor (mCherry) were also obtained with direct excitation for the same fields of view. Approximately 800 FLIM images were thus acquired for each multiwell plate. Such large FRET data volumes require rapid automated analysis, for which we have developed an open source program called *FLIMfit*^35^ (available at http://www.openmicroscopy.org/site/products/partner/flimfit), based on the variable projection method and providing tools for segmentation of cells containing both donor and acceptor, monoexponential and global analysis of EGFP lifetime using convolution and background correction, as well as analysis of fluorescence intensity images.

**Figure 4:**
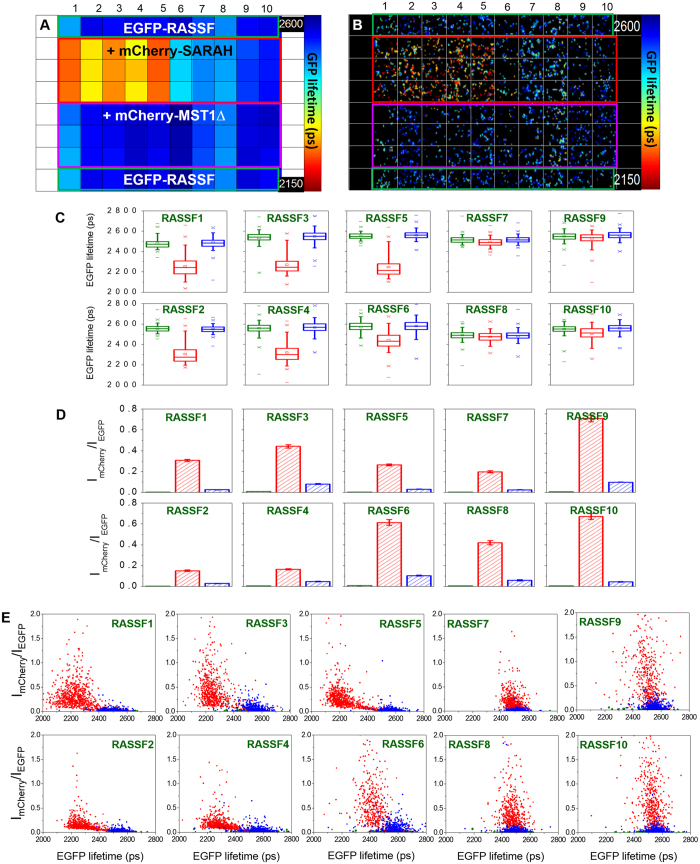
Comparison of the RASSF family members in terms of dimerisation with the SARAH_MST1_ domain using FRET. (**A**) Plate map showing average EGFP donor lifetimes (ps) calculated for 10 fields of view (FOV) per well using a monoexponential fit. (**B**) False-colour FLIM images of cells from a typical FOV in each well showing the EGFP lifetime (ps) per pixel. (**C**) Box plots showing median EGFP lifetimes, interquartile (box range), standard deviation (whisker), 1% and 99% percentile (×) and *minimum/maximum* values (−) calculated for individual cells averaged over 10 FOV per well using monoexponential analysis: green: EGFP-RASSF(1–10) only; red: EGFP-RASSF(1–10) + mCherry-SARAH_MST1_; blue: EGFP-RASSF(1–10) + mCherry-MST1ΔSARAH (see Supplementary material for a table of differences in mean fluorescence lifetime). (**D**) Acceptor/donor intensity ratios (I_mCherry_/I_EGFP_) averaged over each cell for all the conditions in the plate. The colour code is the same as in **C**). (**E**) Scattered plots of EGFP lifetimes versus acceptor/donor intensity ratios (I_mCherry_/I_EGFP_) calculated for individual cells (with same colour code as for **C**). FLIM data were acquired with wide-field imaging.

For these assays, COS7 cells were transfected with donor only plasmids (EGFP-RASSF) and two different conditions of donor plus acceptor constructs: (*i*) EGFP-RASSF + mCherry-SARAH_MST1_ (interaction partner) and (*ii*) EGFP-RASSF + mCherry-MST1ΔSARAH as the negative control (Fig. 4A). Figure 4 shows the results of this screen, displaying the mean donor lifetime fitted to a monoexponential decay profile and averaged across 10 FOV per well. Although we expect to identify two EGFP lifetime components corresponding to free and SARAH-bound RASSF in co-transfected cells, here we show that a monoexponential fit (equation 1) provides a convenient average lifetime value (*τ*) per cell that can be used for qualitative readouts of the occurrence or absence of the protein-protein interaction.


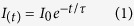


The average EGFP donor lifetimes calculated from the monoexponential pixel-wise fit of all FOV in each of the 96 wells of the RASSF-SARAH plate are displayed as a colour coded plate map (Fig. 4A). Box plots of lifetimes calculated per cell per condition are also shown (Fig. 4C). A montage of FLIM images showing one FOV per well is presented in Fig. 4B.

When cells co-transfected with the negative control (mCherry-MST1ΔSARAH) are compared with those transfected with the donor only, the average of the mean lifetime differences for each RASSF family member was 6 ± 8 ps, with a maximum change in mean fluorescence lifetime of 16 ps for RASSF3. These results represent the biological noise in our measurement. Therefore, to be conservative, we considered that a lifetime shift of at least 32 ps – i.e. twice the maximum difference observed between donor only and donor plus negative control – should be required for it to be considered significant.

In the case of RASSF and SARAH_MST1_ co-transfection, the average EGFP lifetime is reduced by 130–310 ps for RASSF1-6 (Table S.3, Supplementary informationm). This reduction is above our threshold for significance, as outlined above. For RASSF7-10, the reduction of the mean EGFP lifetime in cells co-transfected with SARAH_MST1_ was less than 32 ps, suggesting little or no donor-acceptor interaction (Table S.3, Supplementary information).

The donor and acceptor-labelled proteins used in these experiments were encoded using separate plasmids and therefore they will not be expressed in a 1:1 ratio in the cells (Fig. 4D). Variations in the donor/acceptor ratio do impact the magnitude of the FRET readout and could also impair the significance of the negative controls. For example, if more acceptor molecules are expressed in one cell compared to another, more donor molecules may be quenched and the average donor fluorescence lifetime per cell would then be shorter. In order to clarify this issue, 2D plots of EGFP donor lifetimes versus acceptor/donor intensity ratios have been constructed after segmenting individual cells in all FOVs (Fig. 4E). While a finite spread of the EGFP lifetimes is observed for each condition, the EGFP lifetime distributions for RASSF1-6/SARAH_MST1_ co-transfection only have a small overlap with the distribution of the negative control, the average lifetime being reduced even at low acceptor concentration, as would be expected for FRET. For RASSF7-10/SARAH_MST1_ co-transfection, the EGFP lifetime distributions are centred on similar values as for the negative control, even for high acceptor/donor ratios, indicating a lack of FRET and therefore a weak or no interaction. These 2D plots support the qualitative readouts provided by the average EGFP lifetime obtained from the monoexponential fit of intensity decays that are seen to be robust in the presence of variation in the donor/acceptor stoichiometry. Overall, these results indicate the specific interaction based on the dimerisation of the SARAH domains between RASSF1-6 and MST1, while there is very little or no interaction between the SARAH domain and the coiled-coil or unstructured regions at the C-terminal end of RASSF7-10.

The results of this intracellular FRET assay are supported by biochemical data^15^ and by the SARAH domain heterodimer structural models (depicted in Fig. S.1 in the Supplementary information) showing that most of the main interacting residues of all six SARAH_RASSF_ monomers are well-conserved and aligned to heterodimerise with the SARAH_MST1_ monomer. The contact interface mainly involves the side chains and non-polar residues for all six heterodimers, with a small degree of polar or charged interaction between the acidic and basic residues (see also Table S.1 in the Supplementary information).

Based on these structural models, we selected three key non-polar residues in the main helix for further mutational studies. These are residues that align to L444, L448 and L451 in SARAH_MST1_ and they are highly conserved, as well as major contributors to dimer formation and stability. We applied our FRET screening technique to study the effect of three point mutations within the SARAH domains of RASSF1 and RASSF5C, which are the two best characterised RASSF members with published literature on their L308P and L224A mutants respectively^23^,^36^. All mutations involved the replacement of leucine residues with proline at the positions described above. We chose to perform these mutations since proline residues have been shown to introduce distortions (kinks) to α-helices^37^, in our case in the main helix facilitating the dimerisation with the SARAH_MST1_ domain^26^,^28^.

**Figure 5:**
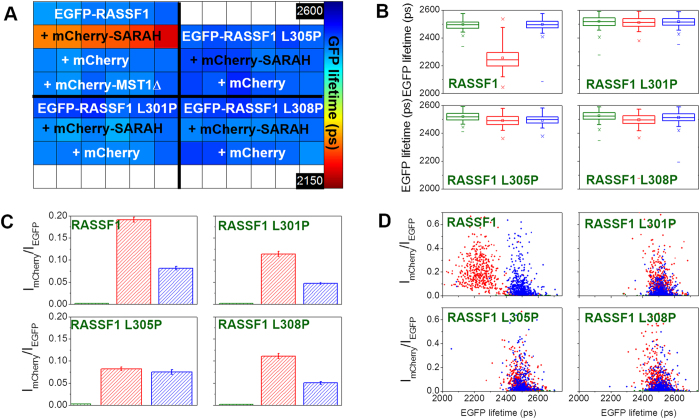
Effect of three different point mutations within the SARAH domain of RASSF1 on the dimerisation with the isolated SARAH_MST1_. (**A**) Plate map showing the average EGFP lifetimes calculated for 10 fields of view per well when fitting to a monoexponential decay profile. The wild-type EGFP-RASSF1 assay shows that mCherry alone can serve as a negative control as well as the mCherry-MST1ΔSARAH. (**B**) Box plots showing median EGFP lifetimes, interquartile (box range), standard deviation (whisker), 1% and 99% percentile (×) and *minimum/maximum v*alues (−) for segmented cells in different conditions within the plate: green: EGFP-RASSF1 (wild type and mutants) only; red: EGFP-RASSF1 (wild type and mutants) + mCherry-SARAH_MST1_; blue: EGFP-RASSF1 (wild type and mutants) + mCherry (see Supplementary material for a table of differences in mean fluorescence lifetime). (**C**) Average acceptor/donor intensity ratios (I_mCherry_/I_EGFP_) for the segmented cells in different conditions within the plate (same colour code as in **B**). (**D**) 2D plots of acceptor/donor intensity ratios versus EGFP lifetimes for the segmented cells in different conditions within the plate (same colour code as in **B**). FLIM data were acquired with wide-field imaging.

**Figure 6:**
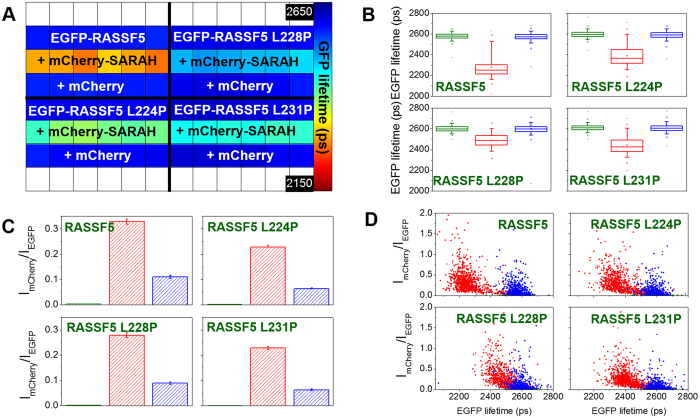
The effect of three different point mutations within the SARAH domain of RASSF5C on the dimerisation with the isolated SARAH_MST1_. (**A**) Plate map showing the average EGFP lifetimes calculated for 10 fields of view per well fitted to a monoexponential decay model. (**B**) Box plots showing median EGFP lifetimes, interquartile (box range), standard deviation (whisker), 1% and 99% percentile (×) and *minimum/maximum* values (−) for the segmented cells in different conditions within the plate: green: EGFP-RASSF5C (wild type and mutants) only; red: EGFP-RASSF5C (wild type and mutants) + mCherry-SARAH_MST1_; blue: EGFP-RASSF5C (wild type and mutants) + mCherry (see Supplementary material for a table of differences in mean fluorescence lifetime). (**C**) Average intensity ratios acceptor/donor (I_mCherry_/I_EGFP_) for the segmented cells in different conditions within the plate (same colour code as in **B**). (**D**) 2D plots of intensity ratios acceptor/donor versus EGFP lifetimes for the segmented cells in different conditions within the plate (same colour code as in **B**). FLIM data were acquired with wide-field imaging.

Figures 5 and 6 show the results of FRET assays of interaction between the wild type isolated SARAH_MST1_ domain and the point mutated RASSF1 and RASSF5 constructs. The average EGFP lifetimes obtained by fitting to a monoexponential decay model indicate that all three point mutations introduced within the SARAH domain of RASSF1 inhibit dimerisation with the isolated SARAH_MST1_ domain (Fig. 5A). The box plots in Fig. 5B indicate that the average values of the EGFP lifetimes show differences of less than 17 ps when co-expressed with the MST1ΔSARAH domain or with mCherry alone as negative controls. The distributions of the EGFP lifetimes versus the acceptor/donor intensity ratios of all RASSF1 mutants overlap with those of the negative control for all acceptor expression levels (Fig. 5D).

In the case of the three RASSF5C mutants, we observe a reduction in the average EGFP lifetimes compared to the negative control based on the box plots in Fig. 6B, suggesting that dimerisation of the SARAH domains still occurs. The distributions on the 2D plots in Fig. 6D are clearly different for the mCherry-SARAH_MST1_ domain co-transfection compared to the co-transfection with mCherry (negative control). However, the reduction in mean EGFP lifetime in individual cells expressing the mutants is smaller than that observed with the wild type RASSF5C, suggesting that the fraction of the bound molecules is reduced in the case of RASSF5C mutants compared to the wild-type protein, which could be due to a reduction in binding affinity.

**Figure 7:**
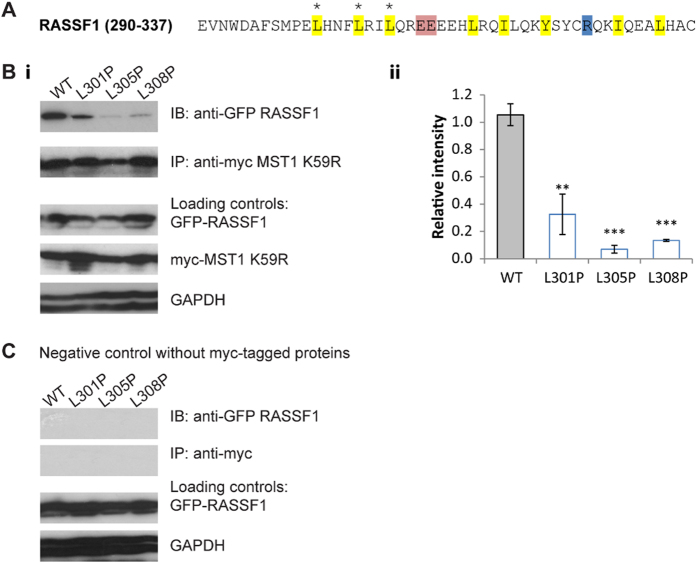
Effects of mutations in the SARAH_RASSF1_ domain on dimerisation with full length MST1. (**A**) The SARAH domain sequence of RASSF1. Main interacting non-polar (yellow), acidic (red) and basic (blue) residues are shown. The three positions in which mutations were introduced are marked by asterisks (*). (**B**) (i) Co-immunoprecipitation assay to show heterodimerisation between myc-MST1 K59R and wild-type (WT) EGFP-RASSF1 and its three mutants. The loading controls are shown below. (ii) Quantification of the bands in terms of relative intensity to the WT control (Mean ± SD. n = 3; *p < 0.05, **p < 0.01, ***p < 0.001). (**C**) Co-immunoprecipitation assay of the negative controls. A simultaneous negative control was performed using cell lysates containing only EGFP-RASSF1 or its mutants. The loading controls are shown below.

**Figure 8:**
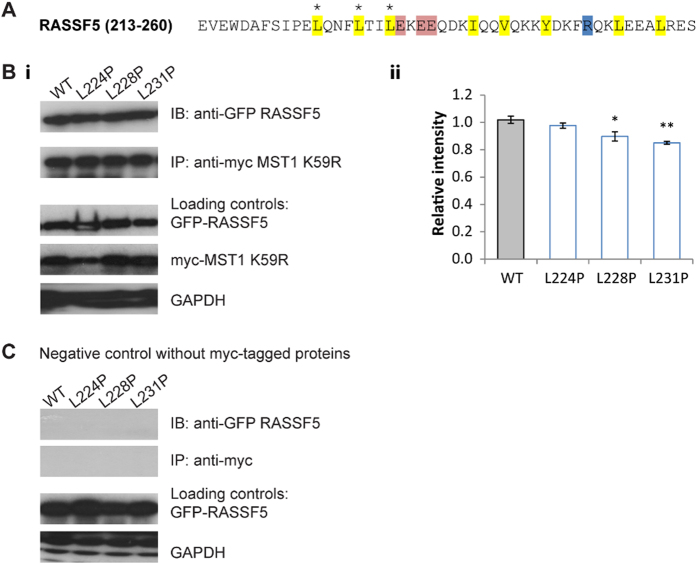
Effects of mutations in the SARAH_RASSF5_ domain on dimerization with full length MST1. (**A**) The SARAH domain sequence of RASSF5. Main interacting non-polar (yellow), acidic (red) and basic (blue) residues are shown. The three positions in which mutations were introduced are marked by asterisks (*). (**B**) (i) Co-immunoprecipitation assay to show heterodimerisation between myc-MST1 K59R and wild-type (WT) EGFP-RASSF5 and its three mutants. The loading controls are shown below. (ii) Quantification of the bands in terms of relative intensity to the WT control (Mean ± SD. n = 3; *p < 0.05, **p < 0.01, ***p < 0.001). (**C**) Co-immunoprecipitation assay of the negative controls. A simultaneous negative control was performed using cell lysates containing only EGFP-RASSF5 or its mutants. The loading controls are shown below.

These FRET screening data are also supported by biochemical assays, represented in Figs 7 and 8, where full length proteins were used to better mimic physiological conditions. For RASSF1 (Fig. 7), only the wild-type protein showed strong binding to MST1, whereas the signals from the mutants were significantly weaker. In the case of RASSF5 (Fig. 8), the mutants were still detected at a significant level, but reduced compared to the wild-type protein. In agreement with the FRET data, these biochemical assays indicate that, while binding still occurs, the affinities are reduced by the mutations.

The different degrees of disruption to heterodimerisation caused by the SARAH mutations in RASSF1 and RASSF5 observed both in our FRET and biochemical data (Figs 5, 6, 7, 8) could be due to distinct biochemical and structural properties of the individual SARAH domains, such as local variations in the specific residues involved in the individual heterodimeric interfaces or in the residues surrounding the mutated sites. It is possible that the neighbouring residues could compensate for the effects of the mutation in RASSF5, but not in RASSF1. Similarly, the leucine residues and hydrophobic interactions may play a more critical role for RASSF1 heterodimerisation compared to RASSF5. Alternatively, the kinks introduced by the proline mutations could affect the secondary structure of SARAH as a whole. It has been shown that the MST1 binding interface increases due to these distortions^28^, so it is plausible that the introduction of proline into RASSF1-SARAH severely distorts its helical structure to the detriment of its ability to dimerise. This effect could be less severe in RASSF5, thus its mutants retain their ability to heterodimerise, albeit at a diminished level.

**Figure 9:**
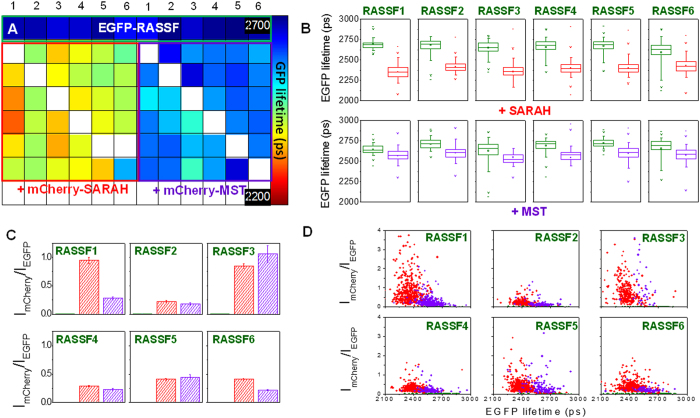
Comparison of the RASSF family members in terms of dimerisation with the isolated SARAH_MST1_ domain and the full length MST1 using FRET. (**A**) Plate map showing average EGFP lifetimes (ps) calculated for 10 fields of view per well by fitting to a monoexponential decay model. (**B**) Box plots showing median EGFP lifetimes, interquartile (box range), standard deviation (whisker), 1% and 99% percentile (×) and *minimum/maximum* values (−) calculated for individual cells from 10 FOV per well; green: EGFP-RASSF(1–6) only; red: EGFP-RASSF(1–6) + mCherry-SARAH_MST1_; purple: EGFP-RASSF(1–6) + mCherry-MST1. (**C**) Average acceptor/donor intensity ratios (I_mCherry_/I_EGFP_) for all the conditions in the plate with the same colour code as in (**C**). (**D**) 2D scatter plots of acceptor/donor intensity ratios (I_mCherry_/I_EGFP_) versus EGFP lifetime calculated for individual cells with same colour code as (**B**). FLIM data were acquired with optical sectioning using Nipkow disc unit.

We also investigated the differences in the binding characteristics between the isolated SARAH domain and the full length MST1 when interacting with those RASSF proteins that are able to dimerise (RASSF1-6). Figure 9A shows the plate map for this experiment. Initially we fitted the donor fluorescence data to a monoexponential decay model, as for the previous assays. The plate map of EGFP donor lifetimes averaged over 10 FOV are shown colour-coded in Fig. 9A together with box plots of the lifetimes on a per cell basis in Fig. 9B. It is immediately apparent that the EGFP lifetimes are more reduced when the RASSF proteins 1–6 interact with the isolated SARAH_MST1_ domain compared to when they dimerise with the full length MST1. This is observed for all RASSF1-6, and is supported by the 2D plots of EGFP lifetimes vs. donor/acceptor ratios (Fig. 9D) even though the donor/acceptor ratios vary among the different conditions within the plate (Fig. 9C).

These data could be explained by a larger mean donor-acceptor FRET distance for the interaction of full length MST1 with RASSF1-6 compared to the interaction with the isolated SARAH domain, e.g. due to steric constraints. This would reduce the FRET efficiency, due to its dependence to the sixth power of the donor-acceptor distance. Alternatively, the fraction of bound molecules, e.g. due to a different binding affinity of RASSF1-6, could be different for the two interactions.

To understand more about the interactions producing the observed differences in the readout based on fitting to the monoexponential decay model, the data underlying Fig. 9 was fitted to a double exponential decay model (equation 2), using the global analysis capabilities of *FLIMfit*^35^.


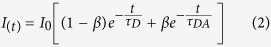


**Figure 10:**
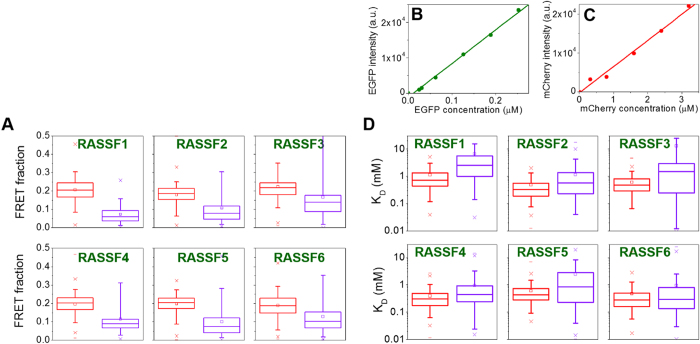
Results of global fitting of the donor fluorescence decay data underlying Fig. 9 to a double exponential decay model. (**A**) FRET population fractions for RASSF1-6 interacting with SARAH_MST1_ (red) and full length MST1 (purple). (**B**,**C**) EGFP and mCherry calibration of intensity versus fluorophore concentration. (**D**) Dissociation constants (K_D_) for RASSF1-6 interacting with SARAH_MST1_ (red) and full length MST1 (purple).

The two lifetime components contributing to the EGFP (donor) decay profiles arise from non-interacting RASSF molecules (unquenched donor, *τ*_*D*_) and from RASSF molecules that are bound either to the isolated SARAH domain or to the full length MST1 kinase (donor quenched by FRET, *τ*_*DA*_). The unquenched donor lifetime was determined using data from the cells transfected only with RASSF1-6 and this component was fixed during the global fitting. The donor lifetime quenched by FRET was allowed to vary, but was constrained to be spatially invariant across all cells within a given experimental condition. Thus, the fraction of interacting molecules (*β* term in equation 2) could be estimated and these results are presented in the box plots shown in Fig. 10A (the full list of parameters obtained from this analysis are presented in the Supplementary information). Owing to the challenges associated with quantifying FRET interactions between fluorescent proteins that are applicable to all such assays^38^, these values should be treated as estimates. Although they show relatively broad distributions, they indicate a lower fraction of bound molecules in the case of the RASSF interaction with the full length MST1 protein compared to the isolated SARAH_MST1_ domain.

To understand if the results in Fig. 10A are indeed due to a difference in binding affinities, we need to estimate the dissociation constants *K*_*D*_ for the interactions of MST1 with the different RASSF1-6 proteins. For this we can use the binding population fractions from the global FLIM analysis, but it is also necessary to estimate the concentrations of the MST1 and RASSF1-6 proteins from the EGFP and mCherry fluorescence intensities. To this end we used the Nipkow disc unit to implement optically sectioned FLIM in order to constrain the detected emission to a well-defined focal volume. The instrument was calibrated using solutions of purified fluorescent EGFP and mCherry at known concentrations in phosphate buffer pH 7.4. Figure 10B,C shows the linear relationship between the average detected fluorescence intensity per pixel and the fluorophore concentration.

Dissociation constants *K*_*D*_ were calculated for each cell assuming a bi-molecular reaction (equation 3), where *D* is the donor-labelled partner, *A* is the acceptor-labelled partner and *DA* is the complex formed by their association:


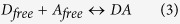


*K*_*D*_ is then given by equation 4, which relates the concentrations of the binding partners to the complex:


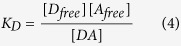


Using the fluorophore concentration calibration we can determine the total donor (*D*_*total*_) and acceptor (*A*_*total*_) concentrations per cell, while the FRET fraction *β* obtained from the global analysis provides an estimate of the concentration of the *DA* complex via the bound fraction of the donor.

We can then write:


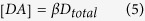



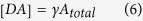


where *γ* is the bound fraction of the acceptor molecules within the complex. This fraction can be calculated from Eq. 5 and 6:


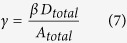


Knowing the bound *D* and *A* fractions, we can obtain the free fractions and re-write the *K*_*D*_ expression:


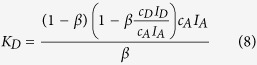


where *I*_*D*_ and *I*_*A*_ are the fluorescence intensities of the donor and acceptor respectively, which are linearly proportional to the concentrations via the proportionality constants *c*_*D*_ and *c*_*A*_ (Fig. 10B,C). A full derivation of this equation is presented in the Supplementary information. To estimate the donor (EGFP) concentration in the cells, the fitted initial intensity of the fluorescence decay (at t = 0) was used rather than the total fluorescence intensity because the FRETing and non-FRETing donors have different brightness due to their different lifetimes and quantum yields.

The *K*_*D*_ values obtained for all the conditions in the plate are plotted in Fig. 10D and are in the same range as previously published values: C. Herrmann and colleagues^39^,^40^,^41^ determined a dissociation constant *K*_*D*_ in the order of hundreds of nM for the RASSF5-MST1 complex in FRET experiments using stopped-flow fluorimetry, while the self-association constant for RASSF5 was found to be 5–10 μM, and that for MST1 was in the low nM range. In their case, *K*_*D*_ was calculated as the ratio between the association rate constant *k*_*on*_ and the dissociation rate constant *k*_*off*_. They measured a higher *k*_*off*_ when full length RASSF5 dimerised with the isolated SARAH domain of MST1 than in the case when dimerisation was performed between the isolated SARAH domains of the two proteins, indicating that the full length proteins have a lower affinity than the isolated dimerising domains. Although in our experiments the *K*_*D*_ values for the isolated SARAH domain and the full length MST1 are not clearly separated (Fig. 10D), there appears to be a trend towards higher average *K*_*D*_ values for the RASSF-full length MST1 interaction, suggesting it has a lower interaction strength than the MST1 interaction with the isolated SARAH domain.

## Conclusions

We have developed a high content assay utilising FLIM FRET to screen for binding partners of MST1 kinase among the RASSF protein family and to quantify the relative interaction affinities. Our custom automated FLIM multiwell plate microscope based on time gated detection is capable of rapid automated image acquisition and therefore facilitates systematic studies of bimolecular processes to provide statistically robust readouts that quickly highlight any systematic errors and effectively average over biological variations. We note that the results presented here and in our previous work^11^,^35^ highlight that the ability to apply global fitting over such large data sets enables us to take advantage of FRET assays with modest lifetime changes (100–200 ps).

We have demonstrated how a relatively simple wide-field FLIM plate microscope can be applied with fitting to monoexponential decay models to provide robust qualitative readouts of FRET, enabling protein interactions to be identified. This is of practical significance since fitting to monoexponential decay models is much less sensitive to system errors such as variations in the instrument response function, compared to fitting to more complex models and there is a wide range of software tools available to fit FLIM data to a monoexponential decay models on a pixel-wide basis. We also note the importance of plotting the ratio of acceptor to donor fluorescence intensities as a function of donor lifetime to elucidate the impact of relative concentrations, e.g. due to variations in transfection efficiency. For more quantitative measurements, the global fitting capabilities of software tools such as *FLIMfit* complement the capacity of the FLIM plate reader to acquire 100’s–1000’s of FOV and permit the population of FRETing donors to be estimated. We have shown that this can be extended to estimate the *K*_*D*_ of protein interactions, which could be used to map systematically signalling networks, providing that the donor and acceptor fluorophore concentrations can be quantified and for this we implemented optical sectioning using a spinning Nipkow disc with our wide-field detection.

The variation in expression levels enabled us to overcome the impossibility of varying the concentrations of the interacting partners within cells in a controlled manner, as usually done when determining *K*_*D*_. By analysing a large number of cells resulting from segmenting hundreds of fields of view, it was possible to obtain data for a range of protein concentrations within a single experiment. We note that for the case of RASSF6, the statistics were less favourable due to relatively fewer cells surviving the transfection process - although the same conditions were applied as for the other RASSF proteins. Thus the data for RASSF6 should be interpreted with particular caution.

The values obtained for the *K*_*D*_ are in reasonable agreement with those obtained in previous experiments utilising different biochemical techniques and report that the binding affinity is lower in the case of heterodimerisation between RASSF proteins and full length MST1 kinase compared to the heterodimerisation of RASSFs with the isolated SARAH domain from MST1. Our experiments thus illustrate the potential to apply automated high content FLIM FRET assays to screen for binding partners and estimate *K*_*D*_ values in cells, which should offer advantage in convenience and biological relevance compared to *in vitro* measurements using purified proteins. To our knowledge, automated FRET-based assays to determine *K*_*D*_ have previously been applied only in solution, either by intensity measurements^42^,^43^,^44^ or by time-resolved measurements of europium luminescence^45^. Previous reports on *K*_*D*_ determination using FRET in cells are limited to intensity-based FRET^46^,^47^, although there is one report of using FLIM to detect FRET and calculate the *K*_*D*_^48^, but these measurements were not implemented in an automated platform to screen protein-protein interactions. Fluorescence correlation spectroscopy has also been used to determine *K*_*D*_^49^,^50^.

We believe that this automated FLIM FRET HCA approach provides a means to screen for protein interactions in their native context that could be scaled to screen large compound libraries. It could also be applied to map cell signalling networks. However, the quantification of the strength of specific interactions does rely on key simplifying assumptions. Below we point out some limitations of the current implementation:

1. The approach here using a simple donor/acceptor FRET pair is applicable to bimolecular interactions, including dimerisation, with a stoichiometry of 1:1. If more than two binding partners interact, e.g. to oligomerise or to form a complex, then FRET could take place between multiple donors and acceptors. The analysis and fitting model would have to be adapted and potentially more complex labelling schemes should be considered, as well as more sophisticated readouts including time-resolved fluorescence anisotropy or parallel measurements of acceptor as well as donor fluorescence. While this would be challenging, we note that three- or four-colour FRET schemes have been implemented using single molecule measurements^51^,^52^ or confocal/multiphoton fluorescence microscopy^53^,^54^. These approaches have been used to study conformational changes in RNA and DNA, multiple protein interactions^55^,^56^ and oligomerisation^57^, although *K*_*D*_ values have not been obtained from such studies. Our current technique could be extended to read out multiple bimolecular interactions within the same or different signalling pathways using multiplexed FRET probes, as we and others have previously shown^58^,^59^.
2. Our approach provides information on the interaction strength between the expressed fluorescently-labelled proteins but one has to consider that, depending on the cell type, the corresponding unlabelled endogenous proteins could also be interacting with the labelled proteins and this would impact the estimates of *K*_*D*_^50^. Most cell-signalling components are expressed at relatively low levels (e.g. compared with housekeeping proteins) and for the COS7 cells used here, we expect the concentration of the endogenous proteins to be 5–10× lower than the corresponding over-expressed labelled protein. Nevertheless, further controls could be implemented in future studies that could include performing experiments in knockout cell lines for proteins of interest or depleting endogenous proteins to verify that this has no effect on *K*_*D*_ estimates. Another approach to overcome this problem would be to label the endogenous proteins using gene editing techniques such as CRISPR/Cas and assay their interactions.
3. Estimations of *K*_*D*_ based on FRET measurements using fluorescent proteins as donor and acceptor fluorophores can be subject to artefacts owing to the uncertainty in the average κ^2^ dipole orientation factor that arise from the fact that the fluorophores do not dynamically randomise their relative orientations during the fluorescence decay^38^, since the rotational correlation time of fluorescent proteins is typically large compared to the excited state lifetime^60^. This can lead to extended FRET efficiency probability distributions that could impact the estimation of the FRETing population fraction and therefore *K*_*D*_. Estimations of the FRETing population fraction can also be impacted by dark acceptor states^38^. These considerations impact all quantitative FRET measurements with fluorescent proteins yet such measurements are widely used and have provided a range of insights into biological processes. If these considerations can be addressed, e.g. by implementing FRET with smaller fluorophores that do result in dynamic averaging of dipole orientation, then the precision and reliability of *K*_*D*_ estimation could be improved.
4. Our estimation of *K*_*D*_ requires knowledge of the absolute concentration of donor and acceptor fluorophores, which we obtain by assuming that the quantum yield of the GFP and mCherry fluorescent proteins is the same in aqueous solution as it is in the cell and that it does not vary significantly throughout the cell. Previous measurements of EGFP report that it presents similar brightness in the cytoplasm and nucleus to what it presents in solution^61^.

The automated FLIM FRET assays reported in this work were undertaken with fixed cells, but could readily be applied to live cells for which similar performance is expected, in line with our previous work^62^. We are developing an open hardware approach to FLIM high content analysis and the latest versions of our open source software for data acquisition and analysis, together with and descriptions of hardware components is available on our website at http://www3.imperial.ac.uk/photonics/research/biomedical-imaging/openflimhca.

## Materials and Methods

### DNA constructs

Full length RASSF1-10 were cloned into the Gateway^®^-modified pEGFP-C1 vector (Clontech) to produce constructs with an N-terminus EGFP tag as described in ref. 15. MST1 (residues 1-487), MST1ΔSARAH (residues 1-431) and MST1-SARAH (residues 432-487) were cloned into pmCherry-C1 vector (Clontech) by restriction digest and ligation at the BglII and HindIII sites. The pTriEx6-MST1 K59R kinase-dead mutant construct used in the biochemical studies has been previously described in ref. 15. All RASSF5 constructs used in this study were derived from the RASSF5C isoform, which has an identical C-terminus region and SARAH domain to RASSF5A.

***Mutagenesis*** was performed using the QuickChange^®^ Site-Directed Mutagenesis Kit (Stratagene) following the manufacturer’s instructions. All mutant constructs were sequence verified.

***Co-immunoprecipitation*** was performed using the Anti-c-Myc Immunoprecipitation kit (Sigma) using co-transfected cell lysates as described in ref. 15. All co-IP assays were repeated three times.

### Western blots

Antibodies used for identification are anti-GFP (B2) (Santa Cruz), anti-GAPDH (Santa Cruz), anti-myc (in-house). Bands from Western blotting were quantified using ImageJ. The relative intensity of the WT control was set at 1 for each experiment and used as a reference point. The error bars are the standard deviations, p-values were calculated using the Student’s t-test and indicated as follows: p ≤ 0.05 (*), p ≤ 0.01 (**) and p ≤ 0.001 (***).

### Protein modelling

Docking programs Hex (http://hexserver.loria.fr/index.php) and ClusPro (http://nrc.bu.edu/cluster) were used for rigid body docking and to run simulations of the heterodimers consisting of the monomer structures of MST1 (PDB: 2JO8) and the RASSF SARAH homology models from ref. 15. Each run generated 100 or more solutions that were ranked by cluster sizes and the top two ranked models were selected and analysed using naccess (http://www.bioinf.manchester.ac.uk/naccess/).

### Cells

COS7 cells (ECACC) were grown in DMEM supplemented with 10% fetal calf serum, 2 mM glutamine, 1 mM sodium pyruvate and 1% penicillin-streptomycin and were used for all experiments. All fluorescent constructs were transfected via electroporation. Typically, 1–1.5 × 10^6^ cells were suspended in 100 μl homemade electroporation buffer (140 mM KCl, 8 mM NaCl, 0.88 mM MgSO_4_, 2.97 mM Na_2_HPO_4_, 1.06 mM NaH_2_PO_4_ and 0.5% (w/v) bovine serumalbumin (pH = 7.4), filtered through a 0.2 μm membrane for sterilisation). 4.5 μg of plasmids in different combinations (indicated in the figures) were added to this suspension. Electroporation was performed using an Amaxa Nucleofector^TM^ II (Lonza, Switzerland) using the manufacturer’s program for COS7 cells. Cells were then seeded in a 96-well plate (Greiner Bio-One) at a density of 30 000 cells/well and fixed the following day using 4% paraformaldehyde for 20 minutes at room temperature, washed 3 times in phosphate buffer saline (PBS) and imaged in PBS.

### Automated FLIM multiwell plate reader

The instrument shown in Fig. 2 was constructed around a motorised Olympus IX 81-Z microscope with ZDC autofocus. The pulsed excitation radiation (60 MHz repetition rate) is selected from the output of a supercontinuum laser (SC 400-6, Fianium Ltd, UK) using band pass filters (Semrock) arranged in a motorised filter wheel.

For wide-field imaging the excitation is directed via a single-mode optical fibre to the back illumination port of the microscope after passing through a rotating diffuser wheel and relayed to the focal plane of the microscope to realise Köhler illumination. The samples arrayed in a 96-well plate were mounted on a motorised x-y stage (Märzhäuser Wetzlar GmbH, Germany) and imaged using a 20 × objective (Olympus UPlanFl 20×/0.5). Appropriate dichroic mirrors and emission filters in the motorised filter cube wheel (GFP: excitation 472/30 nm, dichroic 495 nm, emission 520/35 nm; mCherry: excitation 545/30 nm, dichroic 570 nm, emission 610/75 nm) provided automated selection of spectral channels. The emitted fluorescence light was imaged via the left-hand port of the microscope to a gated optical intensifier (GOI) (Kentech Instruments Ltd., UK) and the resulting gated images at the phosphorus screen were imaged to a cooled CCD camera (Orca ER II, Hamamatsu, Japan). The GOI gating voltage signal is synchronised and delayed with respect to the laser excitation pulses under computer control. For the work reported here, the GOI gate width was set to 1 ns and typically time-gated images of EGFP fluorescence were acquired at 5 different delays after excitation while only one time gated image (at the beginning of the decay) was acquired for the mCherry emission. The integration time of the CCD camera was set to 1–2 s per gate delay for EGFP and 5–6 s for the mCherry image acquisition such that the dynamic range of the CCD was utilised.

For the optically sectioned FLIM acquisitions used to provide the data for the *K*_*D*_ calculations, the instrument was configured to incorporate a spinning Nipkow disk unit (CSU-X1 Yokogawa Electric Corporation, Japan), as described in refs 63, 64, 65, with a 40× air objective (Olympus, LUCPLFLN 40) with an NA of 0.6. The pulsed excitation was directed via a polarisation-preserving single mode optical fibre to the input of the spinning Nipkow disk unit and the fluorescence image was relayed onto the GOI where the time-gated images were acquired as for the wide-field configuration.

In addition to the time-gated FLIM FRET data, a FLIM acquisition of a scattering sample detected at the excitation wavelength was acquired to provide an instrument response function (IRF) for the data analysis. Time-gated FLIM was also applied to a well containing only PBS in order to determine the time varying background.

### FLIM data acquisition and analysis

The instrument is controlled using a programme written in LabVIEW (National Instruments, USA). This controls the automatic movement of the stage, the autofocusing of each field of view, the automatic change of the excitation filters, of the filters and dichroics in the filter cube wheel, the objective lens change, the GOI gating and the CCD camera acquisition. A “prefind” scan was implemented to image the well plate using fluorescence intensity to identify and localise cells and to acquire donor and acceptor intensity images. Specific fields of view in various wells were selected for subsequent FLIM after applying an intensity threshold. FLIM data analysis was performed using the custom written open source software, *FLIMfit*, described in detail in ref. 35 and freely available at www.openmicroscopy.org/site/products/partner/flimfit. For the work reported here we utilised the following capabilities of *FLIMfit*: cell segmentation based on donor and/or acceptor intensity; calculation of average fluorescence intensity of donor and acceptor per cell; fitting the donor fluorescence intensity decays to monoexponential and to double exponential decay models (including instrument response function (IRF) and time-varying background correction); global fitting of donor fluorescence intensity decays across multiple fields of view and wells; visualisation of FLIM data (including rendering of plate maps showing mean EGFP decay times per well and images of one field of view per well). To utilise the relatively small changes in donor lifetime that we have obtained in the FLIM FRET assays reported here from fits to double exponential decay models, it is critical to minimise fluctuations in the IRF and to account for any residual variation. This was realised by acquiring FLIM data of a reference dye solution (rhodamine 6G) in some of the plate wells and fitting the measured decay data to a monoexponential model in order to precisely determine the relative excitation time (i.e. the start of the decay profile, *t*_*0*_), for each plate. This information was combined with the measurement of a scattering sample to construct the IRF that is convolved with the exponential decay model to provide the function to which the experimental FLIM data is fitted. Graphs of lifetime and intensity ratio parameters were plotted in Origin 8 (OriginLab, USA).

## Additional Information

**How to cite this article**: Margineanu, A. *et al*. Screening for protein-protein interactions using Förster resonance energy transfer (FRET) and fluorescence lifetime imaging microscopy (FLIM). *Sci. Rep.* **6**, 28186; doi: 10.1038/srep28186 (2016).

## Supplementary Material

Supplementary Information

## Figures and Tables

**Figure 1:**
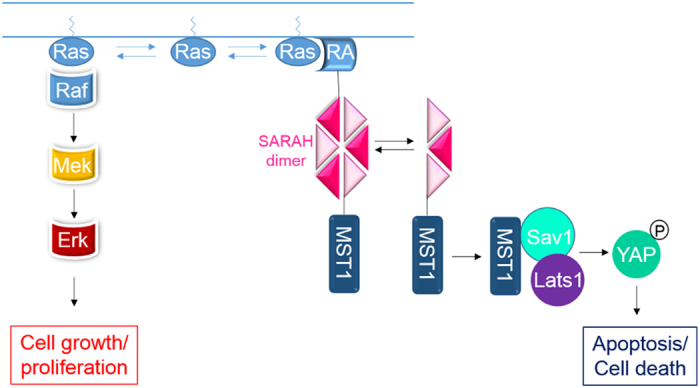
Schematic of Ras-dependent pathways determining cell fate.
