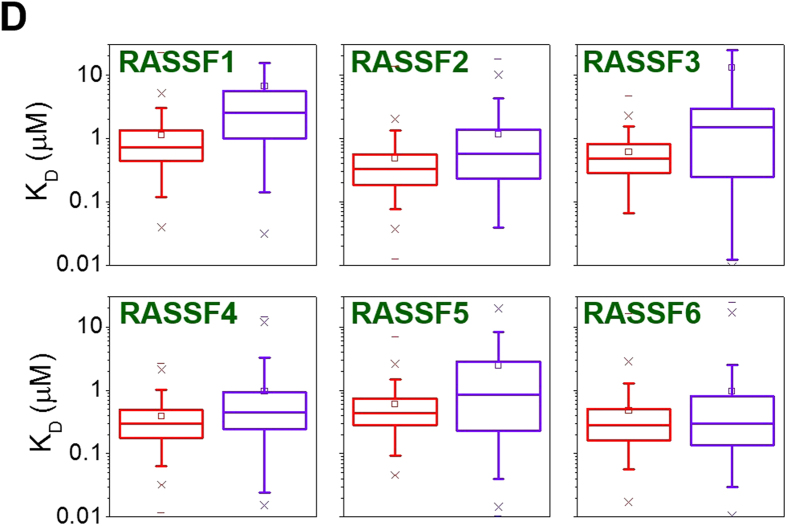# Corrigendum: Screening for protein-protein interactions using Förster resonance energy transfer (FRET) and fluorescence lifetime imaging microscopy (FLIM)

**DOI:** 10.1038/srep33621

**Published:** 2016-09-22

**Authors:** Anca Margineanu, Jia Jia Chan, Douglas J. Kelly, Sean C. Warren, Delphine Flatters, Sunil Kumar, Matilda Katan, Christopher W. Dunsby, Paul M. W. French

Scientific Reports 6: Article number: 2818610.1038/srep28186; published online: 06 24 2016; updated: 09 22 2016

This Article contains an error in Figure 10D, where the y-axis ‘K_D_ (micromolar)’ is incorrectly given as ‘K_D_ (millimolar)’. The correct Figure 10D appears below as Figure 1.

## Figures and Tables

**Figure 1:**