# Supplementary material for: Screening for protein-protein interactions using Förster resonance energy transfer (FRET) and fluorescence lifetime imaging microscopy (FLIM)
Source: Sci Rep. 2016 Jun 24;6:28186. doi: 10.1038/srep28186 (PMC4919659; doi:10.1038/srep28186)
Supplement: Supplementary Information [file srep28186-s1.pdf]

Supplementary information

for the paper:

Screening for protein-protein interactions using Förster resonance energy transfer (FRET) and fluorescence lifetime imaging microscopy (FLIM)

Anca Margineanu[1], Jia Jia Chan[2**], Douglas J. Kelly[1,3**], Sean C. Warren[1,3**], Delphine Flatters[4], Sunil Kumar[1], Matilda Katan[2], Christopher W. Dunsby[1], Paul M.W. French[1*]*

[1]Imperial College London, Dept. Physics, Photonics Lab., Blackett building, Prince Consort Road, London, SW7 2AZ, UK

[2]University College London, Institute of Structural and Molecular Biology, Darwin building, Gower St., London, WC1E 6BT, UK

[3]Imperial College London, Institute of Chemical Biology, London, SW7 2AZ, London, UK

[4]Université Paris Diderot, Sorbonne Paris Cité, Molécules Thérapeutiques in silico, Inserm UMR-S 973, 35 rue Helene Brion, 75013 Paris, France

*To whom the correspondence should be addressed:

Anca.Margineanu@imperial.ac.uk

paul.french@imperial.ac.uk

**These authors contributed equally to this work

SARAH domain contact interface/ interaction mode

Figure S.1*. Heterodimeric models of the SARAH$_{MST1}$ and SARAH$_{RASSF}$ domains. A)* *Multiple sequence alignments of the Prosite predicted SARAH domain. The secondary structural elements are indicated as helix 1 (H1) and helix 2 (H2). Non-polar residues that are critical for the hydrophobic framework are marked with asterisks (*). Important residues involved in dimerisation are also highlighted according to their properties: non-polar (yellow), acidic (red) and basic (blue).* ***B)*** *The structure of the MST1 monomer is in purple and the RASSF monomer in cyan. Non-polar (yellow), acidic (red) and basic (blue) side chains of the residues involved in the heterodimeric interface are shown.*

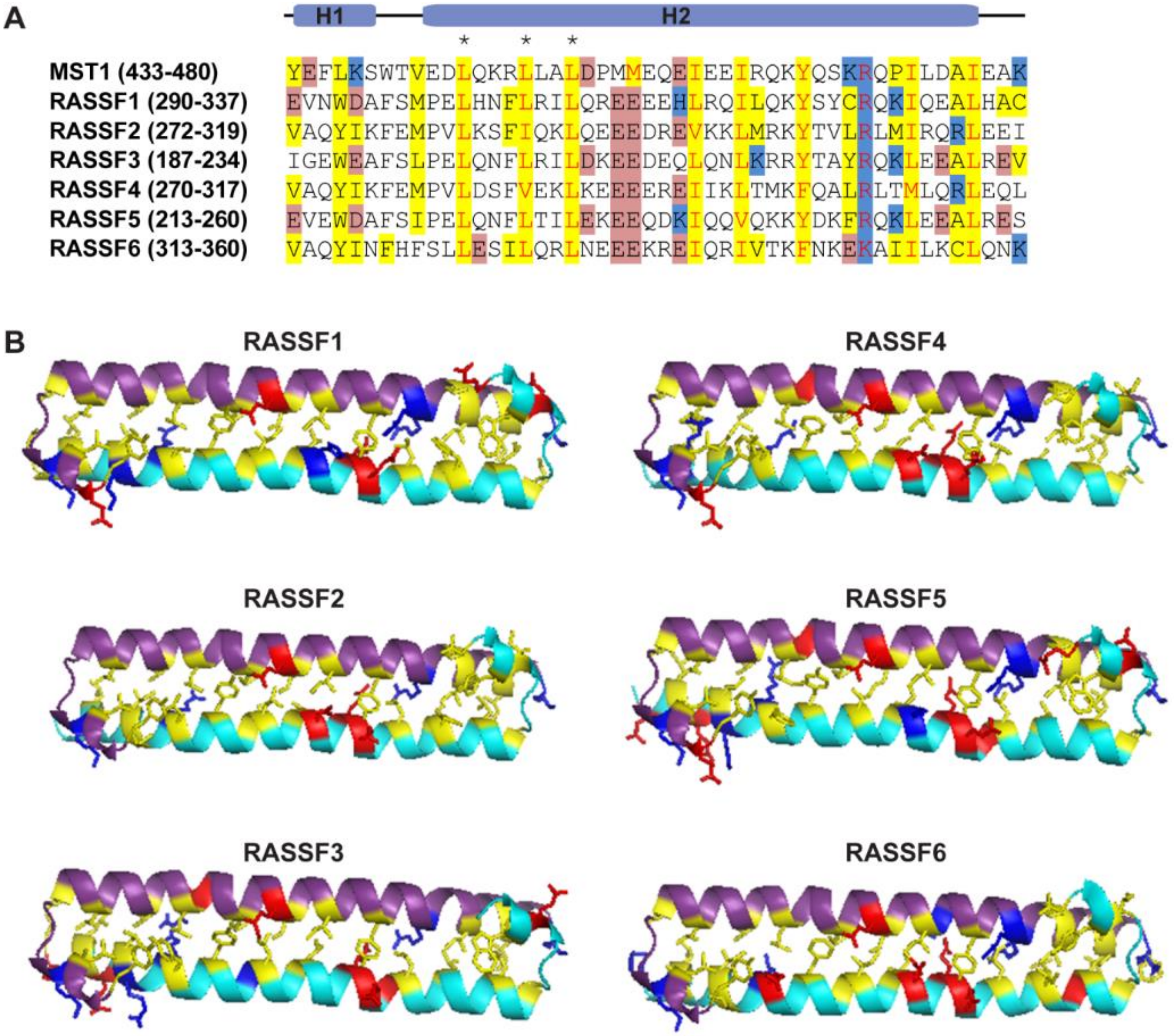

Table S.1 Interface sizes (in Å^2) calculated using *naccess* for the best SARAH heterodimer model of each RASSF with MST1.

MST1 with:	Total	Side chain	Main chain	Non-polar	Polar
RASSF1	2844.6	2733.1	111.6	2350.6	494.1
RASSF2	2933.6	2808.9	124.6	2426.5	507.1
RASSF3	2767.7	2684.2	83.5	2235.5	532.2
RASSF4	2843.2	2731.5	111.7	2296.2	547.1
RASSF5	2820.9	2717.3	103.5	2278.6	542.2
RASSF6	2988.4	2889.5	98.9	2502.1	486.3

Double exponential analysis of the plate measured with the Nipkow disk configuration (sectioned)

For the double exponential analysis, the donor lifetime in the absence of FRET (τ_D) is calculated by averaging the values of the EGFP lifetimes obtained in the wells containing the donor only, while the values of the donor in the presence of the acceptor (τ_{DA}) are obtained by global analysis after fixing the donor only lifetime. These values are listed in the table S.2.

Based on the donor only lifetime τ_D and the lifetime of the donor in the presence of the acceptor τ_{DA}, the FRET efficiency E_{FRET} can be calculated, from which the donor-acceptor distance r is typically estimated in FRET experiments given that the Förster radius R_0 is known for the specific fluorophores pair:

$$E = 1 - \frac{\tau_{DA}}{\tau_D} = \left(\frac{R_0}{r+R_0}\right)^6 \qquad \text{Eq. S1}$$

The R_0 estimation is generally done with the assumption that the orientation angles between the donor and acceptor molecules are randomly distributed. This is considered to be true in the case of small dye molecules that can undergo fast rotation on time scales shorter than the fluorescence lifetime. It has been recently demonstrated that in the case of fluorescent proteins this assumption cannot hold because their rotational correlation times (15-20 ns) are much longer that their fluorescence decay time (2-4 ns) (Vogel S.S., Nguyen T.A., van der Meer B.W., Blank P.S. The impact of heterogeneity and dark acceptor states on FRET: Implications for using fluorescent protein donors and acceptors. *PLoS ONE* **7**, e49593, 2012). Thus, the fluorescent proteins are constrained to a given orientation during the FRET measurements, and the orientation factor (depending on the angle between the two dipoles) must be taken into account as well as the donor-acceptor distance when calculating E_{FRET}.

As it is very difficult to measure experimentally the orientation factor, Vogel et al. (Vogel S.S., van der Meer B.W., Blank P.S. Estimating the distance separating fluorescent protein FRET pairs. *Methods* **66**, 131-138, 2013) proposed an empirical relation to determine *r* in the case of fluorescent proteins, and we have used this reference to obtain the values shown in table S.2.

Table S.2. EGFP lifetimes obtained from double exponential analysis, FRET efficiencies E_{FRET} and donor-acceptor distances *r* for the RASSF-SARAH$_{MST1}$ and RASSF-full length MST1 interactions.

	SARAH				MST1			
	τ_D (ps)	τ_{DA} (ps)	E_{FRET}	*r (nm)*	τ_D (ps)	τ_{DA} (ps)	E_{FRET}	*r (nm)*
**RASSF1**	2632	781	0.703	3.8	2632	889	0.662	4.0
**RASSF2**	2682	956	0.644	4.1	2682	1081	0.597	4.3
**RASSF3**	2660	716	0.731	3.7	2660	505	0.810	3.2
**RASSF4**	2690	865	0.678	3.9	2690	907	0.663	4.0
**RASSF5**	2685	805	0.700	3.8	2685	694	0.741	3.6
**RASSF6**	2688	688	0.744	3.6	2688	705	0.734	3.7

Derivation of the equation for K_D calculation

Dissociation constants K_D were calculated for a bi-molecular reaction as described by equation S1, where *D* is the donor-labelled binding partner, *A* is the acceptor-labelled binding partner and *DA* is the complex formed by their association:

$$D_{free} + A_{free} \leftrightarrow DA \qquad \text{Eq. S1}$$

K_D is then given by equation S2, which relates the concentrations of the binding partners to the complex.

$$K_D = \frac{[D_{free}][A_{free}]}{[DA]} \qquad \text{Eq. S2}$$

Using the fluorophore concentration calibration (figure 10B,C in the text of the paper), we can determine the total donor (D_{total}) and acceptor (A_{total}) concentrations, while the FRET fraction β obtained from the

FLIM global analysis provides an estimate of the concentration of the *DA* complex via the bound fraction of the donor.

We can then write:

$$[DA] = \beta D_{total} \quad \text{Eq. S3}$$

$$[DA] = \gamma A_{total} \quad \text{Eq. S4}$$

where γ is the bound fraction of the acceptor molecules within the complex. From the equality of Eq. S3 and S4, this fraction can be determined:

$$\gamma = \frac{\beta D_{total}}{A_{total}} \quad \text{Eq. S5}$$

Knowing the bound *D* and *A* fractions, we can obtain the free fractions:

$$[D_{free}] = (1 - \beta)\, D_{total} \quad \text{Eq. S6}$$

$$[A_{free}] = (1 - \gamma)\, A_{total} = \left(1 - \frac{\beta D_{total}}{A_{total}}\right) A_{total} \quad \text{Eq. S7}$$

Replacing Eq. S6 and S7 in the K_D expression, we obtain:

$$K_d = \frac{[D_{free}][A_{free}]}{[DA]} = \frac{(1-\beta)\, D_{total}\left(1-\frac{\beta D_{total}}{A_{total}}\right)A_{total}}{\beta D_{total}} = \frac{(1-\beta)\,(1-\beta\frac{c_D I_D}{c_A I_A})c_A I_A}{\beta} \quad \text{Eq. S8}$$

where I_D and I_A are the initial fluorescence intensity of the donor and the acceptor respectively, which are linearly proportional to the donor and acceptor concentrations via the proportionality constants c_D and c_A (as shown in figure 10B,C in the text of the paper).

Tables of changes in donor fluorescence lifetime

Tables S.3-S.5 compare the difference in EGFP lifetime between RASSF proteins interacting with $SARAH_{MST1}$ and the negative control (MST1ΔSARAH or mCherry).

Table S.3. Differences in mean fluorescence lifetimes between RASSF1-10 interacting with $SARAH_{MST1}$ and the negative control (MST1ΔSARAH) (same data as presented in figure 4 in the text of the paper).

	Mean difference in EGFP lifetime (ps)
RASSF1	233
RASSF2	244
RASSF3	279
RASSF4	248
RASSF5	314
RASSF6	138
RASSF7	25
RASSF8	6
RASSF9	12
RASSF10	27

Table S.4. Mean lifetime differences between RASSF1 mutants interacting with $SARAH_{MST1}$ and with the negative control (mCherry) (data presented in figure 5 in the text of the paper).

	Mean difference of EGFP lifetime (ps)
RASSF1	240
RASSF1 L301P	2
RASSF1 L305P	5
RASSF1 L308P	17

Table S.5. Mean lifetime differences between RASSF5 mutants interacting with $SARAH_{MST1}$ and with the negative control (mCherry) (data presented in figure 6 in the text of the paper).

	Mean difference of EGFP lifetime (ps)
RASSF5	294
RASSF5 L224P	201
RASSF5 L228P	109
RASSF5 L231P	165

Examples of dissociation constants K_D for homo- and heterodimerisation of RASSF and MST proteins from literature data

The following data have been taken from references 26 and 39-41, as numbered in the text of the paper. NORE1 is the alternative name for RASSF5. The numbers between brackets represent the aminoacid sequence that was used in the study. Point mutations (e.g. $L^{444}P$) are also indicated. Abreviations: NMR = nuclear magnetic resonance, ITC = Isothermal titration calorimetry, SFF = stopped-flow fluorimetry, CD = circular dichroism, CPM = 7-diethylamino-3-(4'maleimidylphenyl)-4-methylcoumarin, FM = fluorescein-5-maleimide, eCFP = enhanced cyan fluorescent protein, eYFP = enhanced yellow fluorescent protein.

Table S.7. K_D values for NORE1 (RASSF5) and MST1 homo- and heterodimerisation published in literature.

Protein1	Protein2	K_D (μM)	Method	Ref.
RASSF5 SARAH (366-413)	MST1 SARAH (432-480)	Low nM range	NMR	26
NORE1 SARAH (369-413)	NORE1 SARAH (369-413)	34.4 ± 16.4 (at 25°C)<br>154.5 ± 20.5 (at 30°C)	ITC	39
MST1 Inhibitory+SARAH (330-487) $L^{444}P$	MST1 Inhibitory+SARAH (330-487) $L^{444}P$	34.1 ± 4.6 (at 25°C)	ITC	39
NORE1 RBD+SARAH (199-413) $C^{220}S/R^{242}C$-CPM	NORE1 RBD+SARAH (199-413) $C^{220}S/R^{242}C$-FM	3.8	FRET in SFF	39
NORE1 RBD+SARAH (199-413) $C^{220}S/R^{242}C$-CPM	NORE1 RBD+SARAH (199-413) $C^{220}S/C^{414}$-FM	4.7	FRET in SFF	39
NORE1 RBD+SARAH (199-413) $C^{220}S/C^{414}$-CPM	NORE1 RBD+SARAH (199-413) $C^{220}S/C^{414}$-FM	9.8	FRET in SFF	39
NORE1 RBD+SARAH (199-413) $C^{220}S/R^{242}C$-CPM	NORE1 SARAH (369-413) $C^{220}S/C^{414}$-eYFP	3.1	FRET in SFF	39
NORE1 RBD+SARAH (199-413) $C^{220}S/C^{414}$-CPM	NORE1 SARAH (369-413) $C^{220}S/C^{414}$-eYFP	8.3	FRET in SFF	39
MST1 Inhibitory+SARAH (330-487) $F^{487}C$-CPM	MST1 SARAH (437-487)-eYFP	Low nM range	FRET in SFF	39
MST1 Inhibitory+SARAH (330-487) $E^{460}C$-CPM	MST1 Inhibitory+SARAH (330-487) $E^{460}C$-FM	Low nM range	FRET in SFF	39
NORE1 SARAH (369-413)-eCFP	MST1 SARAH (437-487)-eYFP	0.2	FRET in SFF	39
NORE1 SARAH (369-413)-eCFP	MST1 Inhibitory+SARAH (330-487)-eYFP	0.6	FRET in SFF	39
NORE1 RBD+SARAH (199-413) $C^{220}S/C^{414}$-CPM	MST1 SARAH (437-487)-eYFP	0.7	FRET in SFF	39
NORE1 SARAH (369-413)-eCFP	MST1 SARAH (437-487) $L^{444}P$-eYFP	0.6	FRET in SFF	39
MST1 SARAH (437-487)	MST1 SARAH (437-487)	1.07 ± 0.7	CD	40

MST1 SARAH<br>(437-487)	MST1 SARAH<br>(437-487)	0.9 ± 0.1	ITC	40
NORE1 SARAH<br>(370-413)	NORE1 SARAH<br>(370-413)	28	CD	41
